# An Image Analysis Algorithm for Malaria Parasite Stage Classification and Viability Quantification

**DOI:** 10.1371/journal.pone.0061812

**Published:** 2013-04-23

**Authors:** Seunghyun Moon, Sukjun Lee, Heechang Kim, Lucio H. Freitas-Junior, Myungjoo Kang, Lawrence Ayong, Michael A. E. Hansen

**Affiliations:** 1 Image Mining (IM) Group, Institut Pasteur Korea, Seongnam-si, Gyeonggi-do, South Korea; 2 Malaria Drug Discovery (MRA) Unit, Institut Pasteur Korea, Seongnam-si, Gyeonggi-do, South Korea; 3 Center for Neglected Diseases (CND3), Institut Pasteur Korea, Seongnam-si, Gyeonggi-do, South Korea; 4 Department of Mathematics, Seoul National University (SNU), Gwanak-Gu, Seoul, South Korea; Université Pierre et Marie Curie, France

## Abstract

With more than 40% of the world’s population at risk, 200–300 million infections each year, and an estimated 1.2 million deaths annually, malaria remains one of the most important public health problems of mankind today. With the propensity of malaria parasites to rapidly develop resistance to newly developed therapies, and the recent failures of artemisinin-based drugs in Southeast Asia, there is an urgent need for new antimalarial compounds with novel mechanisms of action to be developed against multidrug resistant malaria. We present here a novel image analysis algorithm for the quantitative detection and classification of *Plasmodium* lifecycle stages in culture as well as discriminating between viable and dead parasites in drug-treated samples. This new algorithm reliably estimates the number of red blood cells (isolated or clustered) per fluorescence image field, and accurately identifies parasitized erythrocytes on the basis of high intensity DAPI-stained parasite nuclei spots and Mitotracker-stained mitochondrial in viable parasites. We validated the performance of the algorithm by manual counting of the infected and non-infected red blood cells in multiple image fields, and the quantitative analyses of the different parasite stages (early rings, rings, trophozoites, schizonts) at various time-point post-merozoite invasion, in tightly synchronized cultures. Additionally, the developed algorithm provided parasitological effective concentration 50 (EC50) values for both chloroquine and artemisinin, that were similar to known growth inhibitory EC50 values for these compounds as determined using conventional SYBR Green I and lactate dehydrogenase-based assays.

## Introduction

Malaria remains one of the most widespread infectious diseases of mankind, with 40% of the world’s population at risk and more than 240 million infections each year [1]. Recent estimates indicate a staggering mortality rate of over 1.2 million deaths annually, of which the majority are children and pregnant women living in sub-Saharan Africa [1], [2]. At least five different *Plasmodium* species are known to cause malaria in humans, the most virulent being *P. falciparum* [3]. Unfortunately, efforts aimed at controlling the disease globally are often confounded by lack of vaccines and the propensity of malaria parasites to rapidly develop resistance to newly developed drugs [4]. There is, therefore, a continuous need for new antimalarial drugs with novel mechanisms of action (MoA) to be developed against multidrug resistance in malaria.

The parasite life cycle includes two asexual replication cycles in humans (exo-erythrocytic or liver forms, and erythrocytic or blood forms) and one sexual reproduction cycle in the mosquito vector [5]. In the human host, *P. falciparum* preferentially infects mature and enucleated red blood cells (RBC), where they develop through a ring and trophozoite stage, and then undergo three to four rounds of DNA synthesis, mitosis and nuclear division to produce a syncytial schizont with approximately 12 to 40 nuclei per infected RBC [5], [6]. This asexual cycle occurs over a period of 48 hours resulting in the release of over 16 merozoites (1n) for additional rounds of host cell invasion and parasite proliferation. These processes of trophic development, schizogony, egress, and host cell invasion represent unique opportunities for the development of new drugs with novel MoA against the infection. However, finding novel drug candidates targeting the parasite’s developmental cycle remains a challenge, as all current antimalarial screening assays mostly rely on total DNA measurements or detection of various parasite proteins as a measure of parasite viability in vitro [7]–[9].

Automated image acquisition technology and computerized image mining techniques can provide multi-parametric and highly accurate information on parasite responses to different drugs during experimentation. Here, we describe a novel and fully automated image analysis algorithm for high content screening (HCS) of drugs against malaria, with the capability of discriminating between living and dead parasitaemia from *in vitro* blood samples. Additionally, we will show that the algorithm is capable of classifying the different life cycle stages of malaria parasites for the purpose of assessing the effect of promising antimalarial compounds on schizont development, parasite egress and host cell invasion processes. Such a system will facilitate the current efforts aimed at identifying new antimalarial drugs, and vaccines targeting the different erythrocytic stages of *P. falciparum* parasites.

## Materials and Methods

### Malaria Parasite Culture, Image Acquisition Process and Image Analysis Algorithm

**Figure 1:**
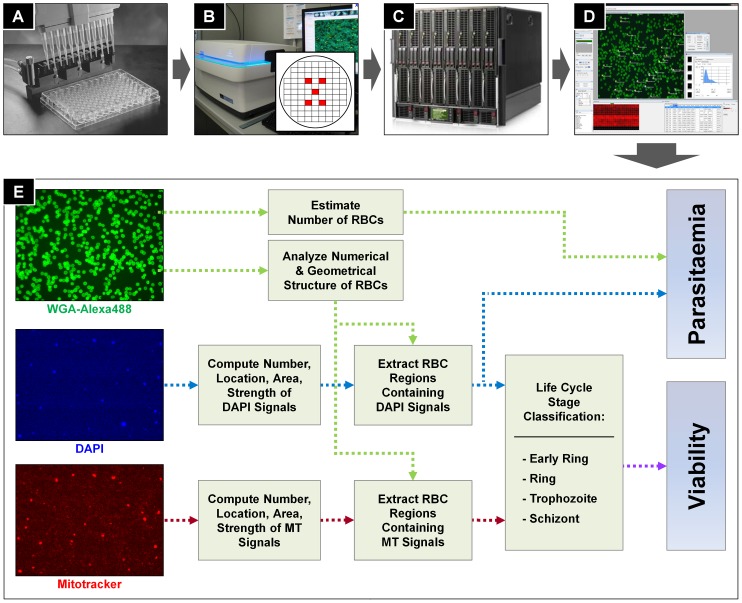
Illustration of the HCS system and the malaria image analysis algorithm. (A) 384-well screening platform to set up experimental assays. (B) Operetta 2.0 imaging system to acquire image data. (C) Central database to store acquired image data. (D) IM platform to load and analyze image data from central database. (E) The malaria image analysis algorithm diagram from input image data to analysis results. The algorithm is implemented as a plug-in of IM platform.

Our HCS system (illustrated in Figure 1) consists of the following five parts: an automated 384-well screening platform to set up the experimental assay, an Operetta™ harmony 2.0 imaging platform (PerkinElmer) to acquire the images, a central database server to store the acquired image data and final analysis results, an image analysis software platform, and finally the malaria image analysis algorithm designed as plug-in’s to the IM platform.

The experimental assay was set up using the following protocol: *P. falciparum* strain 3D7 (MRA-102) was obtained from the Biodefense and Emerging Infections (BEI) research resources (Manassas, VA) and maintained in human RBC (blood type O^+^, Gyeonggi Blood Center, Korean Red Cross) at 4% hematocrit in media consisting of RPMI 1640, 25 mM HEPES buffer (pH 7.4), 0.1 mM hypoxanthine, 0.016 mM thymidine, 0.5% Albumax, and 20 µg/ml gentamycin. Cultures were grown at 37°C in 75-cm^2^ flasks after gassing with a mixture of 5% CO_2_, 1%O_2_, and 94% N_2_. When needed, parasites were double synchronized (8-hour interval) at the ring stage by sorbitol-treatment and further cultivated through one complete cycle prior to each assay. Drug-treated and control cultures were diluted to 0.01% hematocrits (representing at least 0.03% parasitaemias) in a staining solution comprising wheat germ agglutinin-AlexaFluor488 conjugate (RBC stain), DAPI (Invitrogen D3571, nuclei stain), and Mitotracker Red CMXRos (Invitrogen M7510, active mitochondrial stain) each at a 1 nM concentration. The cultures in 384-well glass plates (Matrical) were then incubated for 20 minutes at 37°C to allow for complete incorporation of each dye prior to image acquisition.

Following the parasite and red blood cell staining as described in the previously (Figure 1A), five microscopic image fields (red boxes of the grid in Figure 1B) were acquired from each well using an automated Operetta 2.0 imaging system (Figure 1B). The images were taken using 40× lens and the dimension of each image is 1360×1024 pixel^2^ ( = 340×256 *µ*m^2^). In real-time, the acquired images were transferred to a central database and stored as TIFF image format with a bit depth of 16 bits (unsigned integer, values ranging from 0 to 65535) (Figure 1C). We used an in-house developed (proprietary) image analysis software platform called Image Mining (IM) which is able to access the central database and serve as an interface between the acquired image data and the dedicated image analysis algorithm (Figure 1D). The malaria image analysis algorithm was developed and implemented as a ‘plug-in’ to the IM platform. Although, the used platform is proprietary, any other image processing software (Matlab™, ImageJ, etc.) is fully capable of analyzing the image data, following the in-here described algorithm.

Input images are composed of three different channels for WGA-AlexaFlour488 fluorescence (green), DAPI fluorescence (blue), and for Mitotracker fluorescence (red). Figure 1E is a diagram of the malaria image analysis algorithm, from the input image data to the analysis results. The algorithm is designed to determine the proportion of infected RBC (parasitaemia), the proportion of each life cycle stage (early ring, ring, trophozoite, or schizont), and the proportion of live versus dead parasite populations at each developmental stage. The algorithm consists of four major parts: 1) RBC number estimation, 2) parasite signal detection, 3) infected RBC detection, and finally 4) life cycle stage classification.

### Total Red Blood Cell Number Estimation Process

The total number of RBCs per image field represents the most important parameter for accurately determining culture parasitaemia in malaria. The parasitaemia is defined as the ratio of the number of infected RBCs to the total number of RBCs. RBCs in microscopic images often appear as clustered corpuscles and the most challenging part to count the total number of RBCs is when RBCs are clustered together. To estimate the total number of RBCs on Giemsa-stained glass slides, most of the previous research have used a detailed segmentation approach to separate clustered RBCs into individual RBCs such as use of region growing [10]–[12], morphology [13], [14], graph [15], contour tracing [16], distance transform [10], [17], or template matching [18], [19]. Although, these methods result in an estimate of total RBCs, they are extremely time consuming, and consequently increase the execution time significantly. In a HCS setup, most of these methods are not applicable and hence another approach has to be found.

**Figure 2:**
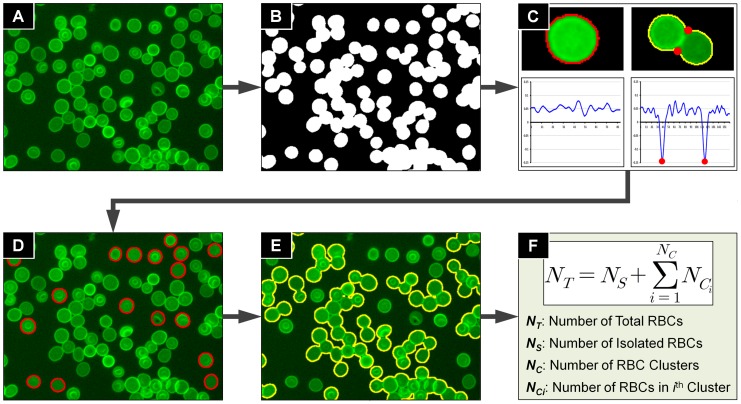
Total RBC number estimation process. (A) A partial region of 400×300 pixel^2^ of WGA-AlexaFlour 488 fluorescence channel image. (B) Separate RBC regions by Otsu’s threshold method. (C) Classify isolated and clustered RBCs by concave corner point detection method based on boundary curvature measurement and principal axes ratio. (D) Count number and calculate average area of isolated RBCs. (E) Estimate number of RBCs in each cluster. (F) Compute number of total RBCs.

On average, the number of RBCs in each image varies between 450 and 650, and the observed proportions of the number of RBCs in clusters are 80∼90% of the total number of RBCs (Figure 2A). And the maximum parasitaemia is below 10%. Thus, as mentioned previously, if we use a ‘the detailed segmentation approach’, a lot of computation time is required to split clusters, and >90% of the cost for the segmentation of the clusters will be spent for no other reason than to measure the number of RBCs. In order to accurately estimate the total number of RBCs per image field, and limit the image processing time necessary for HTS experimentations, we have developed and validated a novel approach for cell number estimation in various RBC clusters. We identified the following three characteristics with stained RBCs: 1) the overlap area between two adjacent RBCs in a cluster (of two) is in most cases negligible, 2) the area of the single and isolated RBCs are homogeneous and approximately following a normal distribution with (relatively) low variation, and finally, 3) there are enough single and isolated RBCs in each image (10∼20% of the total number of RBCs). Based on these observations, we can compute an average RBC area from the isolated RBCs, and estimate the number of RBCs in each cluster by dividing RBC cluster area by the average RBC area. Later we will show statistics that proves this gives us similar results to manual counting.

Estimating the total number of RBCs is done in five sequential steps: 1) separating RBCs from the background, 2) classifying the isolated and clustered RBCs regions, 3) calculating an average area of the isolated RBCs, 4) estimating the number of RBCs in clustered RBCs, and 5) computing the total number of RBCs per image field by summation of the cell count in all clusters to the number of isolated RBCs. Figure 2 shows the flow diagram of the process.

The first step is to detect and separate the RBC signals from background fluorescence (Figure 2B). Average intensities of backgrounds and RBC regions are around 50±10, and 190±40 respectively. And RBC regions occupy 20∼30% of the total image area. Since background and RBC regions intensity profiles are clearly separable and RBCs are well spread over the entire image domain, simple adaptive threshold methods can be applied to separate RBC regions from background. We used Otsu’s threshold method [20] to automatically eliminate detectable background signals during image processing.

The second step was to classify and split all segmented areas into isolated and clustered RBCs. We employed the following criteria to detect clearly isolated RBCs: 1) the object has no concave corner points, 2) the ratio of the length of major and minor axis of the object is close to 1 (one requirement of a circle shaped object). For the first criterion we used an osculating circle estimation method [21] to detect concave corner points of an object boundary based on the curvature. Let 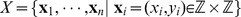 be the chain code [22] of boundary of an object, that is 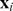 belongs to the boundary and 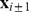 is a neighboring pixel of 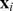. Then, the osculating circle
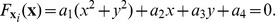
at a boundary point 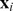 with respect to a given window size 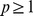 is computed by a least square minimization problem



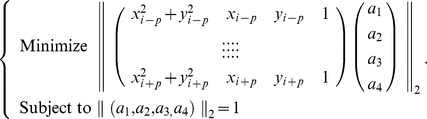



Then the center 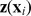 and radius 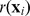 of the osculating circle at 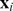 are given by.
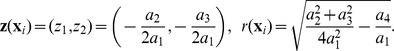
and therefore the local curvature of the boundary at 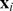 is



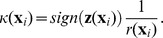
where 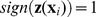 if 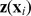 is inside of the object region, otherwise 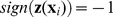.

If a point on the boundary has a negative local minimum curvature, then the point is a concave corner point (Figure 2C). Moreover, if all curvature values of an object boundary points are positive, then the object has no concave (inwards) corner point which satisfies the first criterion. Thus isolated RBC candidates satisfying the first criterion can be classified by scanning the curvatures of the boundary points. The second criterion is made to identify RBC clusters passing the first criterion i.e. having no visible corner points. This type of RBC cluster is usually composed by two RBCs are overlapped over than half and therefore yielding an elongated (ellipsoidal) shape with no or undetectable concave corner point. The malaria image analysis algorithm computes the ratio of the length of major and minor axes which are defined by the longest line and shortest line from a boundary point to another boundary point. If the ratio of an object is close to 1, then the object has circular shape, because there is no object of symmetry shape having no concave corner point except circular cells in the images. If an object satisfies these two criteria, then the object is classified to an isolated RBC (Figure 2D), otherwise the object is classified to a RBC cluster (Figure 2E).

The third step is calculating the average RBC area. Let 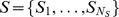 be a set of isolated RBCs with 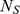 elements classified from the previous step, and 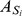 be the area of 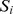. Then an average area of isolated RBCs 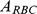 is calculated by
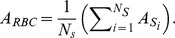



The fourth step is estimating the number of RBCs in RBC clusters. This estimation is achieved by dividing RBC clusters area by 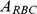. Let 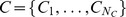 be a set of RBC clusters with 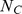 elements and 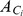 be the area of 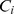. Then the number of RBCs 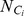 in a cluster 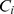 is estimated by
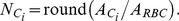



Finally the estimated number of total RBCs 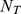 is computed by
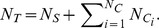



A validation of the RBC number estimation process is given in the results section, by comparing algorithm estimation results to manual counting.

### Parasite Signal Detection Process

**Figure 3:**
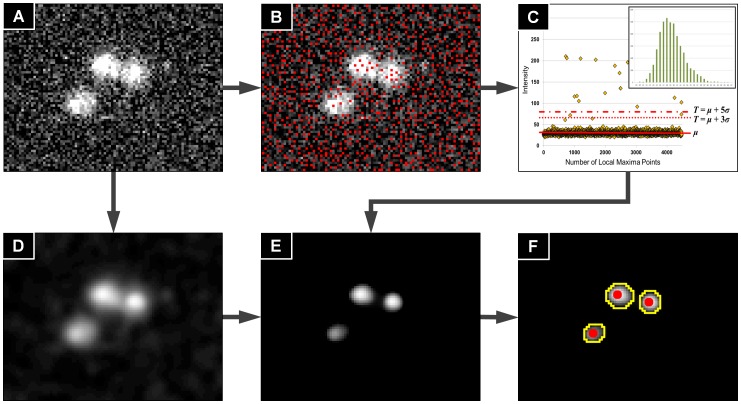
Parasite signal detection process diagram. (A) DAPI or Mitotracker fluorescence channel image. (B) Detect local maxima points of intensity. (C) Compute 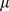 and 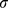 of local maximum point intensities. (D) Smooth signals by Gaussian filtering. (E) Separate signals by threshold value 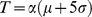. (F) Compute number, location, area, signal strength information.

The malaria image analysis algorithm extracts information about the number of parasites, their location and area and finally their signal strength, by using both the DAPI and Mitotracker fluorescence signals. Figure 3A show a partial image region of size 90×70 pixel^2^ in the DAPI fluorescence channel (rescaled intensity of range [0, 50] from [0, 150] for enhanced visibility). The average background levels of DAPI fluorescence channels have an intensity level between 10 and 20 while the intensities of the parasites are more than 100. Despite the different nature of the DAPI and Mitotracker (DAPI binds to DNA and Mitotracker binds to mitochondria of the parasites), the intensity characteristics of the Mitotracker fluorescence channel images are similar to the ones of the DAPI channel. Therefore the parasite signals in both DAPI and Mitotracker channels can be detected by a same signal detection method.

The parasite signals are detected using a local maxima point detection method. In this process, noise signals on the background are also detected by the method. We observed the intensity distribution of the noise signals from the local maxima points. As shown in Figure 3C, the distribution is close to Gaussian with the average 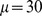 and the standard deviation 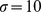, and most of the local maxima points are located within the 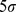 range of 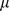. Note that this noise distribution analysis is not influenced from the parasite signals because the number of parasite signal points is smaller (hundreds) than the number of the noise signal points (150,000). Therefore we assume that the parasite signals are outliers of the distribution with signals larger than 99.99994% of the other local maxima points. In conclusion, the parasite signals can be detected by taking signals over than 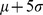.

The parasite signal detection process is done in the following steps: First, the algorithm detects local maxima points of intensity from input images of window size 3×3 pixel^2^ (Figure 3B), and calculates the average 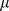 and the standard deviation 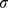 of the detected local maxima points (Figure 3C). Gaussian filtering with 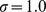 is then applied to the input image to get clear signal regions and suppress the influence of noise (Figure 3D). The parasite signals are then separated from the background by taking pixels with a threshold value 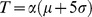 (Figure 3E). Here 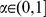 is a constant to compensate the intensity loss due to Gaussian filtering (we fixed the value 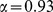 yielding the optimal separation between background spots and parasites). Finally, the algorithm segments each parasite signal by connected component object labeling [23] to the thresholded image, and collects the information of location and area (Figure 3F). For each of the objects, the location of a parasite signal is given by the location of the corresponding local maximum point and the area is given by the number of pixels of the object. A validation of the parasite signal detection process is given in the results section, by comparing algorithm detection results to manual detection.

### Infected Red Blood Cell Segmentation Process

After the parasites have all been identified, the next step is to segment infected RBCs and link each of the parasites to an infected RBC. This is necessary to determine the parasitaemia and parasite viability (dead/alive). Below, we describe the process needed in order to accurately segment RBC clusters and correctly assign the detected parasites to individual RBCs. The following characteristics of human RBCs were exploited to segment the clustered RBCs via circle fitting: 1) RBCs have (approximately) circular shapes with uniform areas, 2) the outer boundaries of RBCs are brighter than its interior regions due to their biconcave nature. On the basis of this latter difference, a RBC edge structure map was generated and a gradient vector field was computed. From here we use a circle fitting model via vector flow tracking to more precisely find the boundary of the RBC.

The Hessian based edge detection filter [24], [25] is used to extract boundary structure from the input image. The filter is designed to detect edge structures with directional information so that it can extract edge structures while suppressing blob objects. Let 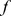 be an input image of RBCs and 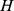 be the Hessian matrix of 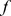 defined by
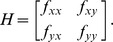



Let 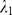, 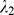 be the eigenvalues of 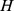, and 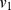, 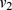 be the corresponding eigenvectors. Without loss of generality, let 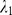 be the eigenvalue that has larger absolute value and 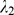 be the eigenvalue with smaller absolute value. Then 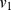 is the direction of greatest curvature (given by the second-order derivative), 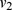 is the direction of least curvature, and the corresponding eigenvalues are the respective amounts of the greatest and least curvatures respectively. The eigenvectors of 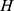 are called ‘principal directions’ and are directions of pure curvature. The eigenvalues of 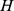 are called principal curvatures and are invariant under rotation. Moreover, 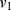 directs from brighter region to darker region if 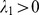 (positive curvature), and directs from darker region to brighter region if 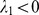 (negative curvature). Based on the properties, a Hessian based edge detection filter 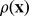 for each pixel 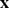 is defined by
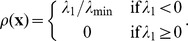
where 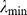 denotes the smallest eigenvalue over all pixels in the image, which in practice will always be smaller than zero [25]. Then 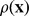 lies in the range of 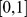, and 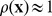 if 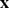 is a pixel on the edge structure, and 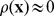 when 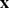 lies on a flat region. In addition, the orientation of the edge for each pixel is given by 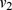. Note that 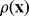 indicates bright edge structures that have negative curvature.

**Figure 4:**
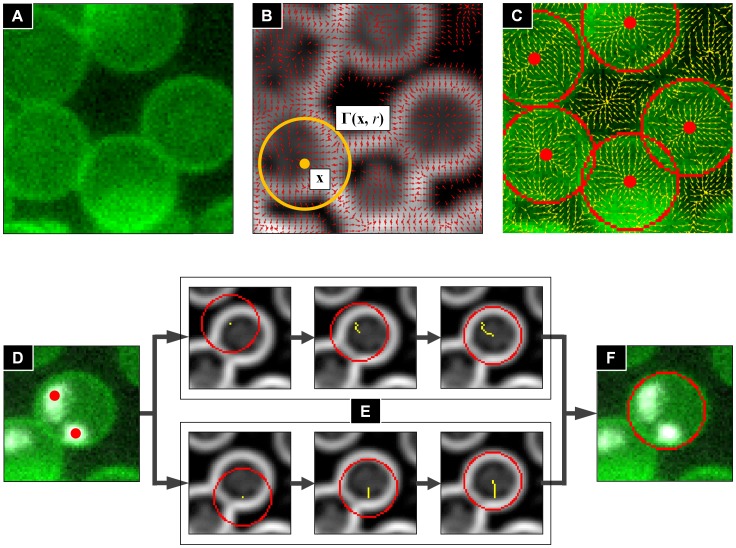
Infected RBC segmentation process diagram. (A) WGA-AlexaFlour488 fluorescence channel image. (B) RBC edge structure map (white structures), corresponding gradient vector filed (red arrows), and the fitting circle 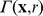 (yellow circle). (C) Normalized vector field of the sum of the gradient vectors along a fitting circle (yellow arrows). (D)-(F) Infected RBC segmentation process. (D) Infected RBC with detected parasite signal points (red dots). (E) Two fitting processes started from different signal points. (F) Fitting result. Inner region of fitting circle is segmented as infected RBC region. Note that two different fitting processes give to same result.

From the RBC edge structure map 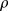
(white structures in Figure 4B), the algorithm calculates the gradient vector field 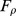 of 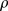 (red arrows in Figure 4B). Let 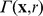 be a fitting circle where the center is 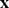, and the radius 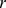 is initiated as the average area of the isolated RBCs calculated from the total RBC number estimation process (yellow circle in Figure 4B). Now we define a vector field 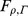 by
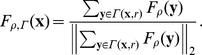



That is, 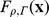 is a normalized vector of the sum of the gradient vectors of 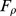 along the direction of 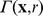. Then as shown in Figure 4C, the vectors in 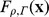 (yellow arrows) are converged toward the RBC centers (red dots). Therefore, the center 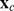 of a RBC can be found by tracking the vector flow of 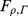 started from any point 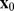 inside the RBC boundary, and then the inner region of 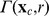 is being the RBC region (red circles in Figure 4C).

The infected RBC segmentation process is done in accordance with the following steps: Firstly, to reduce computational cost, the malaria image analysis algorithm extracts partial image region of size 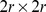 around a parasite signal detected in the previous process where 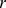 is the average RBC radius calculated from the average RBC area from the total RBC number estimation process (Figure 4D). The RBC edge structure map 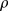 and the gradient vector field 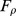 are then computed from the region, and start the fitting process. An initial center point 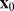 of 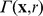 is then set to the location of the detected parasite signal (a red dot of Figure 4D), and 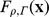 is computed for 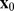. The next point 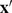 that the current point 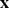 flows through 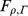 is computed as
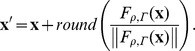
and the angle between 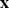 and 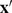 is determined as



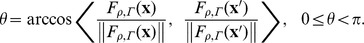



The fitting process is repeated while 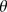 is less than 90 degrees. If 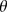 is greater than 90 degrees the fitting process is stopped since a RBC center 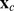 is reached [26].

When the fitting procedure has ended, then the segmented region of 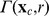 indicated the detection of the RBC, and 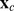 its center. Next, all parasites inside 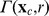 are linked to the (infected) RBC (Figure 4F). Note that the fitting process started from different parasite signals in a same RBC converge to the same result, as shown in Figure 4E. Therefore we can reduce computational cost by doing the fitting process for only one parasite signal among all detected signal points in a same RBC. A validation of the infected RBC segmentation process is given in the results section, by comparing algorithm detection results to manual detection.

### Life Cycle Stage Classification Process

To compose information of infected RBCs, parasite signals detected from DAPI and Mitotracker fluorescence channels are assigned to the segmented RBC as described above. The malaria image analysis algorithm identifies the viable status of the parasites in each infected RBC by the presence of parasite signals detected from the Mitotracker fluorescence channel (Mitotracker parasite signals). The parasites are designated as “alive” if the Mitotracker parasite signals are present in an infected RBC, considered as “dead” if not.

**Figure 5:**
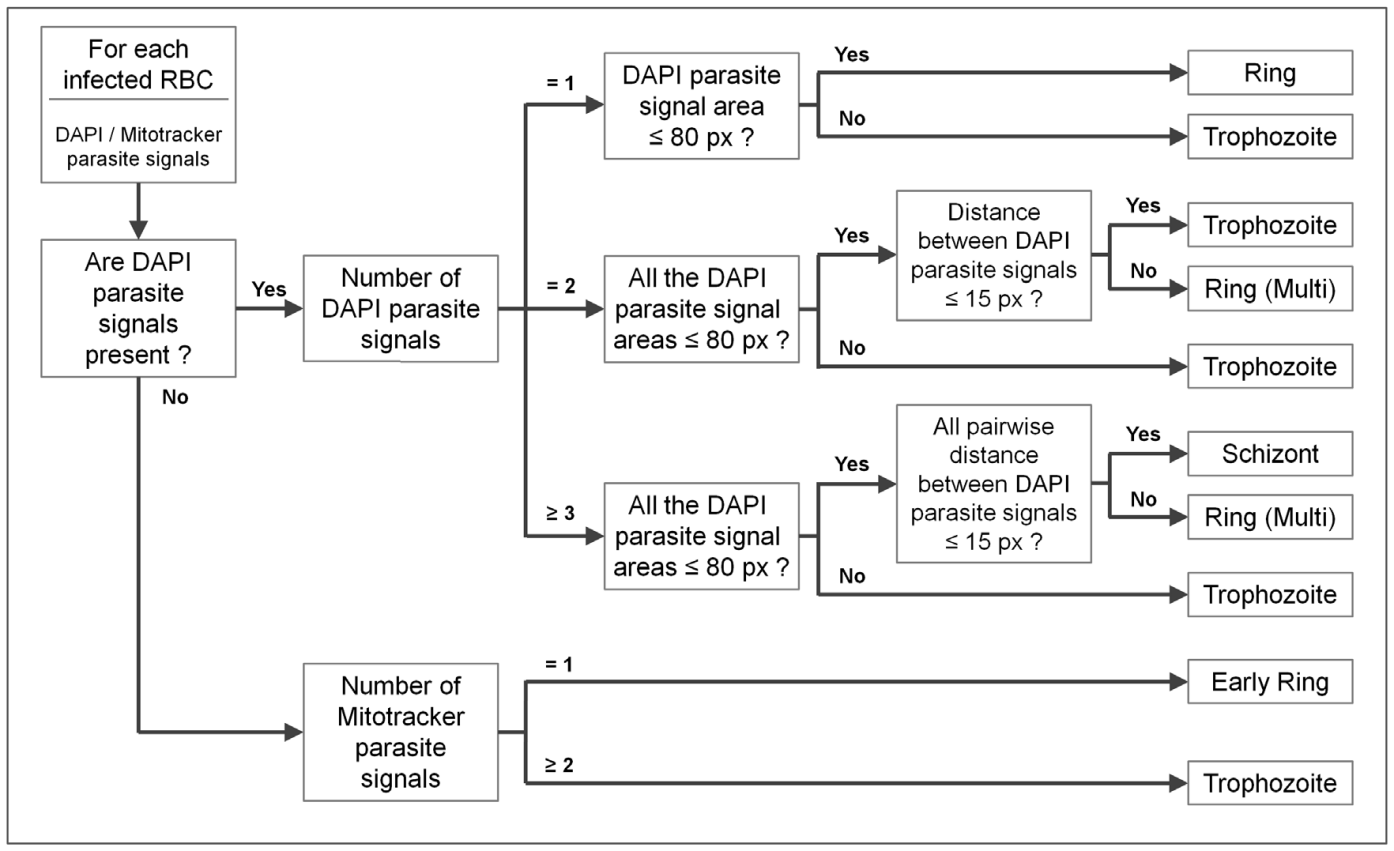
Decision tree of the life cycle stage classification criteria. The life cycle stage of parasites in an infected RBC is classified into early ring, ring, trophozoite and schizont by the presence of the detected parasite signals from the DAPI/Mitotracker fluorescence channels, and the number, area and distance between the signals. Note that “DAPI parasite signal” and “Mitotracker parasite signal” in the decision tree represent the parasite signals detected from the DAPI and Mitotracker channels respectively.

The life cycle stages of the parasites are classified using following criteria which are based on the information of location and area collected from the parasite signal detection process. Figure 5 is the summary of the life cycle stage classification criteria given in the form of a decision tree. If an infected RBC has parasite signals detected from the DAPI fluorescence channel (DAPI parasite signals), then we classify the life cycle stage of the parasites into ring, trophozoite or schizont based on the number, area and distance between the parasite signals. If number of the signals equals to one, then the parasite is: ring stage if the area of the signal is less than or equal to 80 pixels (5 *µ*m^2^), trophozoite stage if not. If the number of the signals is equal to two and the area of each signal is less than or equal to 80 pixels, then the parasites are: ring stage if the distance between signals is greater than 15 pixels (3.75 *µ*m), trophozoite stage if not. If the number of the signals is greater than or equal to three, the area of each signal is less than or equal to 80 pixels, and the maximum pairwise distance between signals is less than or equal to 15 pixels, then the parasites are schizont stage. In this case, living or dead status is decided by presence of the Mitotracker parasite signals as described above. If there exist only Mitotracker parasite signals without DAPI parasite signals (presumably represent parasites at the interphase stage), then we classify the stage of the parasites into two different stages based on the number of the Mitotracker parasite signals in an infected RBC. If number of the signals is equal to one, then the stage is ring, or trophozoite with invisible xsome/uncoiled DNA if two or more. In this case, status of the parasites is live. A validation of the life cycle stage classification process is given in the results section, by using highly synchronized *P. falciparum* parasites at different time-points post infection.

### Parasitaemia and Viability Calculation

Parasitaemia is measured by the ratio of the number of infected RBCs over the total number of RBCs. Here we compute proportions of early ring, ring, trophozoite and schizont stages by the ratio of the number of early ring-infected, ring-infected, trophozoite-infected and schizont-infected RBCs over the total number of infected RBCs. Viabilities of the stages are also calculated by the ratio of the number of early ring-infected, ring-infected, trophozoite-infected and schizont-infected RBCs which have Mitotracker fluorescence signals over the total number of early ring-infected, ring-infected, trophozoite-infected and schizont-infected RBCs. These proportions are important data to find hit compounds which block schizont development in malaria parasites.

## Results

As described above, the validation of the malaria image analysis algorithm was achieved by comparing the obtained data from the RBC number estimation, parasite signal detection, infected RBC segmentation processes with results from manual counting involving six different experimenters. For this purpose, nine image fields from different assay wells were randomly selected and processed using the algorithm and manual annotation. The life cycle stage classification process was validated by applying the algorithm to highly synchronized *P. falciparum* Dd2 cultures at various times post-merozoite invasion. Furthermore, we validated the algorithm for drug EC50 determination by utilizing images from various dose-response experiments with known antimalarial compounds.

### Red Blood Cell Number Estimation Validation

**Figure 6:**
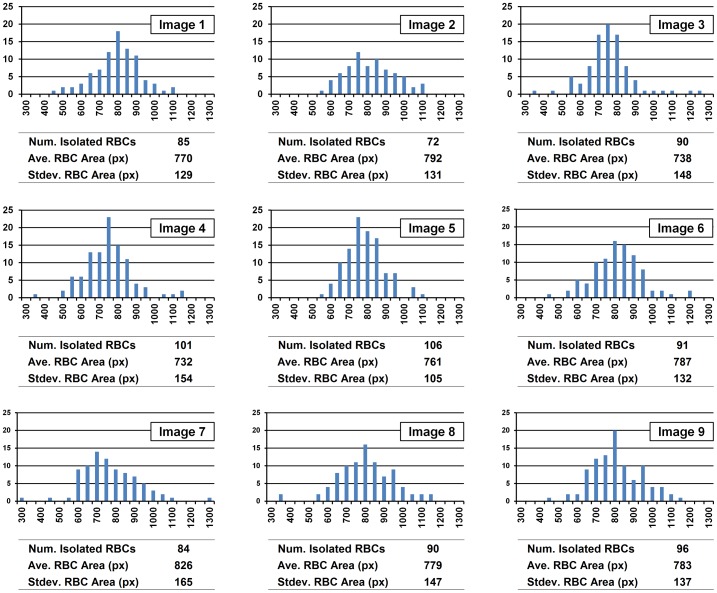
Area distribution and number of isolated RBCs in randomly selected nine images. Each image has enough number of isolated RBCs to calculate average RBC area with distribution close to a normal distribution.

The RBC number estimation process uses an average area of isolated RBCs to estimate number of RBCs in the clusters. Thus it should be validated to have correct results that the stained RBCs images have following characteristics: 1) the overlap area between any two adjacent RBCs in a cluster is in most cases negligible, 2) the areas of the RBCs are uniform and the distribution of the areas approximately follows a normal distribution with low variation, and finally, 3) there are enough number of isolated RBCs in each image. The first characteristic was satisfied by controlling the density of RBCs in experiments. The second and third characteristics were validated by analyzing the area distributions and counting the numbers of isolated RBCs in the nine images. As shown in Figure 6, for each image the distribution forms approximately a normal distribution of 770±140 pixels of RBC area which implies 15.7±1.5 pixels of RBC radius, and there are 72∼106 isolated RBCs which is enough number to estimate average RBC area. Table 1 shows comparison results of manual and algorithm counting of the total number of RBCs in the nine images. Variability of manual counting is maximum 3%, and the errors between manual and algorithm counting are less than 5%. That means manual counting results are reliable and the RBC number estimation method is also robust and reliable.

**Table 1:** Comparison results of manual and algorithm RBC counting.

Image No.	1	2	3	4	5	6	7	8	9	Total
MC 1*	495	549	594	533	617	494	499	590	612	4983
MC 2	523	541	603	546	622	497	515	581	612	5040
MC 3	504	562	617	572	632	504	519	605	639	5154
MC 4	506	556	624	573	637	501	514	595	626	5132
MC 5	490	535	598	542	618	483	491	580	607	4944
MC 6	498	570	622	575	639	486	493	568	602	5053
MC Ave	503	552	610	557	628	494	505	587	616	5051
Algorithm	489	528	620	552	606	498	495	578	617	4983
Error	2.79%	4.58%	1.67%	0.88%	3.55%	0.77%	2.05%	1.47%	0.11%	1.36%

* MC means manual counting result.

### Parasite Signal Detection Validation

The parasite signal detection process was validated by manual inspection of detected signals from the malaria image analysis algorithm. The algorithm detected parasite signals from DAPI and Mitotracker fluorescence channels that are located in RBC regions only. Table 2 shows the validation results for the nine randomly selected images. As shown in the error row, the average errors of parasite signal detection results from both DAPI and Mitotracker fluorescence channels were <2%, and all the false positive and the false negative were occurred in regions where several parasites gathered closely. Additionally, the errors of parasite signal detection results from the DAPI fluorescence channels were larger than the Mitotracker fluorescence channels because the noise signals in DAPI fluorescence channels were stronger than the noise signals in Mitotracker fluorescence channels. In conclusion, the parasite signal detection process is robust and reliable.

**Table 2:** Inspection results of parasite signal detection.

	DAPI				Mitotracker			
	Manual	Algorithm	F.Pos*	F.Neg*	Manual	Algorithm	F.Pos	F.Neg
Image 1	8	8	0	0	5	5	0	0
Image 2	51	53	2	0	24	24	0	0
Image 3	60	59	0	1	29	28	0	1
Image 4	8	9	1	0	1	1	0	0
Image 5	7	7	0	0	20	20	0	0
Image 6	69	67	1	3	26	27	1	0
Image 7	10	11	1	0	6	6	0	0
Image 8	15	15	0	0	6	6	0	0
Image 9	75	75	1	1	31	31	0	0
Total	303	304	6	5	148	148	1	1
Error	-	0.33%	1.98%	1.65%	-	0.00%	0.68%	0.68%

* F.Pos and F.Neg mean false positive and false negative respectively.

### Infected Red Blood Cell Segmentation Validation

The infected RBC segmentation process was also validated by manual inspection of the segmented RBC regions from the malaria image analysis algorithm. Table 3 shows the validation results for the nine randomly selected images. Note that the number of infected RBCs in an image may be different from a number of detected parasite signals in Table 2 because several parasite signals can be located in an infected RBC. In the columns, the false positive means that the malaria image analysis algorithm segmented non-infected RBCs, the false negative means that the algorithm missed infected RBCs, and the misaligned means that the algorithm detected infected RBCs with incorrect regions. As shown in Table 3, the average error of the algorithm was around 2% of misaligned, with no false positive or false negative. Note that since the infected RBC segmentation process uses the parasite signal detection results as starting points of the fitting process, there may be not false positive or false negative of infected RBCs as long as the parasite signal detection results is robust.

**Table 3:** Inspection results of infected RBC segmentation.

	Manual	Algorithm	False Positive	False Negative	Misaligned
Image 1	5	5	0	0	0
Image 2	22	22	0	0	1
Image 3	20	20	0	0	0
Image 4	5	5	0	0	0
Image 5	19	19	0	0	0
Image 6	21	21	0	0	0
Image 7	11	11	0	0	0
Image 8	15	15	0	0	1
Image 9	30	30	0	0	1
Total	148	148	0	0	3
Error	-	0.00%	0.00%	0.00%	2.03%

### Life Cycle Stage Classification Validation

**Figure 7:**
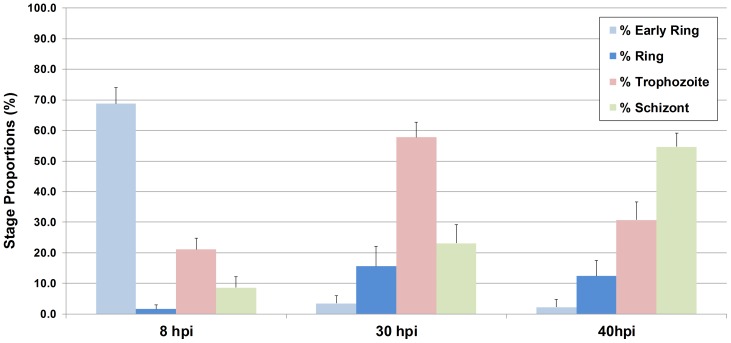
Life cycle stage classification process validation result. Highly synchronized *P. falciparum* Dd2 cultures were imaged at 8, 30 and 40 hpi and were analyzed by the malaria image analysis algorithm. The dominant parasite stages at 8, 30, 40 hpi were early rings, trophozoites, schizonts respectively.

We used highly synchronized *P. falciparum* parasites at different time-points post infection (8, 30 and 40 hours post-infection, hpi) to validate the life cycle stage classification process. *P. falciparum* Dd2 was synchronized repetitively with gelatin and sorbitol method [27]. Tightly synchronous *P. falciparum* Dd2 were seeded at early ring stage (∼8 hpi) and imaged immediately or at 30 or 40 hpi as described above. At each time point, 200 images (5 image fields in 40 wells) were acquired for further processing using the malaria image analysis algorithm. Figure 7 shows the life cycle stage classification validation results. Dd2 cultures were accumulated at particular stages depending on the time post-infection, indicating stage-specific classification capability of the algorithm. As expected, the dominant parasite stages at 8, 30, 40 hpi were early rings, trophozoites, schizonts, respectively.

### EC50 Determination using the Malaria Image Analysis Algorithm

**Figure 8:**
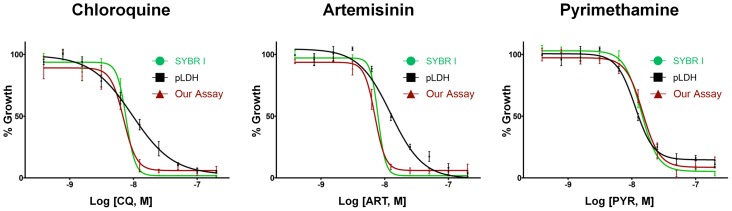
Comparison of EC50 for known antimalarial compounds. EC50 values of the chloroquine, artemisinin, and pyrimethamine against P. falciparum 3D7 parasites using SYBR I, pLDH, and our assays were determined and compared.

We determined the EC50 values for the antimalarial compounds chloroquine, artemisinin, and pyrimethamine against *P. falciparum* 3D7 parasites using the SYBR I and pLDH assays in comparison to our approach. Tightly synchronized cultures at the early ring stage were incubated for 72 hours with each compound and assayed by each test method. For all our assays, drug EC50 values were determined on the basis of culture parasitaemia as determined by the malaria image analysis algorithm. As shown in Figure 8 and Table 4, obtained EC50 values from each assay were similar indicating high correlation between the different assays. On the basis of the parasitaemia from positive growth cultures and the uninfected RBC wells, we calculated Z’ factor as following equation:
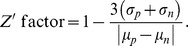
with the means 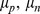 and standard deviations 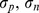 of the positive (*p*) and negative (*n*) controls respectively. The assay Z' factors were 0.784 for our assays, 0.724 for SYBR I, and 0.558 for the pLDH. Together, these results demonstrate high reliability of the new assay for use in high-content drug susceptibility studies.

**Table 4:** Comparison of EC50 for known antimalarial compounds.

	EC50 against *P. falciparum* 3D7 (72-h treated)		
	SYBR I	pLDH	Our assay
Chloroquine	7.69±2.2 nM	9.55±3.1 nM	7.01±2.6 nM
Artemisinin	7.88±1.3 nM	12.47±2.3 nM	7.04±2.3 nM
Pyrimethamine	13.89±1.3 nM	12.27±4.2 nM	14.78±2.6 nM

## Discussion

In this article we have presented a fully automated image analysis algorithm for HCS based antimalarial drug discovery and proved its robustness. The aim of the algorithm is to accurately determine malaria culture parasitaemias and viabilities, and quantify the proportion of each parasite stage per infected RBC. This is achieved by unbiased detection and enumeration of total RBCs, the DAPI-stained parasite nuclei and also Mitotracker-stained mitochondria in viable parasites.

The algorithm computes the total number of RBC from the WGA-AlexaFlour488 channel, which forms the base information to estimate the parasitaemia. Also numerical and geometrical structures of the RBC in the channel can be estimated by the algorithm to be used in the infected RBC detection process. From the DAPI and Mitotracker fluorescence channels, the algorithm quantitatively detects the fluorescence signal of within each parasite, extracting information on number, location, area, and signal strength of fluorescent spots. The algorithm determines infected RBCs based on the location of the detected parasite signals from DAPI and Mitotracker fluorescence channels, and the infected RBC regions are segmented from their numerical and geometrical structures analyzed from the WGA-AlexaFlour488 fluorescence channel. The parasite classification in early rings, rings, trophozoites, or schizonts are then determined following criteria. Finally, the algorithm outputs the results mentioned above by combining all the collected information as tabular format.

Approaches such as region growing [10]–[12], morphology [13], [14], graph [15], contour tracing [16], distance transform [10], [17], or template matching [18], [19] have been employed to estimate RBC numbers in malaria glass slides and/or in microtiter plates. However, such approaches are incapable of precisely determining the number of cells in RBC clusters that are often encountered during HTS experimentation. To accurately and rapidly quantify RBC numbers in the image fields, we employed a novel approach that is based on the mean area of isolated RBCs per image field. As demonstrated by the manual counting validation, our customized image analysis approach is sufficiently precise in determining the total RBC numbers per image field, and offers the added advantage of high processing speed when compared to individual segmentation methods. The parasite signal detection process provides basic information of the parasites states which is used in the infected RBC segmentation and parasite life cycle stage classification processes. The infected RBC segmentation process is applied only for the infected RBCs in order to segment RBC regions, and therefore the computational cost of the algorithm is much less than entire RBC segmentation methods. The parasite life cycle stage classification process provides key information of viabilities of different life cycle states. The parasitaemias are computed by the ratio of number of infected RBCs and total RBCs. The viabilities of different life cycle stages are computed by the ratio of the number of infected RBCs of live parasites and total infected RBCs.

Precise determination of culture parasitaemia and parasite viability is especially necessary to detect and prioritize highly effectiveness drug candidates with novel MoA for development. Additionally, as shown by our time-point stage detection and quantification data (Figure 7), the developed algorithm accurately quantifies each parasite stage in cultures. This potential of our algorithm could be employed to characterize various drugs cellular mechanisms of actions including the drug effects on schizont development, egress and host cell invasion. Taken together, our data demonstrate that the newly developed the image analysis approach is adequately robust for use in HTS antimalarial assays, or as secondary assays for hit validation and confirmation studies. To our knowledge, our image analysis approach represent the first high-content imaging system capable of quantitatively determining the effects of antimalarial drugs on parasite viability and life cycle progression. The utility of this newly developed algorithm in characterizing the antimalarial activities of various small molecules has recently been demonstrated in a high content screen of various chemically diverse compound libraries (manuscript in preparation).
